# Decreased Functional Connectivity of Brain Networks in the Alpha Band after Sleep Deprivation Is Associated with Decreased Inhibitory Control in Young Male Adults

**DOI:** 10.3390/ijerph20054663

**Published:** 2023-03-06

**Authors:** Jie Lian, Lin Xu, Tao Song, Ziyi Peng, Xinxin Gong, Jie Chen, Xiao Zhong, Xin An, Shufang Chen, Yongcong Shao

**Affiliations:** School of Psychology, Beijing Sport University, Beijing 100084, China

**Keywords:** sleep deprivation, inhibitory control, event related potential, resting state, functional connectivity

## Abstract

Sleep deprivation leads to reduced inhibitory control in individuals. However, the underlying neural mechanisms are poorly understood. Accordingly, this study aimed to investigate the effects of total sleep deprivation (TSD) on inhibitory control and their neuroelectrophysiological mechanisms from the perspective of the time course of cognitive processing and brain network connectivity, using event-related potential (ERP) and resting-state functional connectivity techniques. Twenty-five healthy male participants underwent 36 h of TSD (36-h TSD), completing Go/NoGo tasks and resting-state data acquisition before and after TSD; their behavioral and electroencephalogram data were recorded. Compared to baseline, participants’ false alarms for NoGo stimuli increased significantly (*t* = −4.187, *p* < 0.001) after 36-h TSD. ERP results indicated that NoGo-N2 negative amplitude increased and latency was prolonged (*t* = 4.850, *p* < 0.001; *t* = −3.178, *p* < 0.01), and NoGo-P3 amplitude significantly decreased and latency was prolonged (*t* = 5.104, *p* < 0.001; *t* = −2.382, *p* < 0.05) after 36-h TSD. Functional connectivity analysis showed that the connectivity of the default mode and visual networks in the high alpha band was significantly reduced after TSD (*t* = 2.500, *p* = 0.030). Overall, the results suggest that the negative amplitude increase in N2 after 36-h TSD may reveal that more attention and cognitive resources are invested after TSD; the significant decrease in P3 amplitude may indicate the impairment of advanced cognitive processing. Further functional connectivity analysis indicated impairment of the brain’s default mode network and visual information processing after TSD.

## 1. Introduction

Total sleep deprivation (TSD) is defined as occurring when an individual remains awake for a period of at least one night and generally for at least 24 h [1]. In contemporary society, TSD has become very common among certain professionals who sometimes have to work long shifts without breaks, including health workers, long-distance bus drivers, pilots, and rescue workers. TSD can impair an individual’s cognitive function, leading to occasionally serious accidents [2]. Studies have shown that TSD leads to declines in a range of cognitive processes, including attention, alertness, emotion regulation, learning, and memory [3,4,5,6]. The most important of these is the decline in attention; such decline has the most severe effects. In this regard, Gibbings (2021) [7] suggested that one night of TSD significantly reduces an individual’s alertness and attention.

In addition, numerous studies have verified that TSD impairs individuals’ inhibitory control [8], defined as an individual’s efforts to exclude interference from attentional processing and inhibit inappropriate behaviors or dominant responses, thereby exhibiting flexibly to change [9]. The Go/NoGo (GNG) task has been widely used to study the neurobiological mechanisms of inhibitory control [10]. This task randomly presents two different letters or patterns alternately, asking participants to respond to one of the stimuli (called the Go task) and not to the other (called the NoGo task). Incorrect responses to NoGo stimuli are often considered an indicator of response cessation difficulties.

Previous researchers have identified two important electroencephalogram (EEG) components of the GNG paradigm: NoGo-N2 and NoGo-P3. NoGo-N2 is a negative component engaged approximately 200–400 ms after the NoGo stimulus, reflecting monitored conflicting information and the allocation of attentional resources to facilitate subsequent response inhibition [11]; it thus represents the early stage of response inhibition [12]. Meanwhile, NoGo-P3, the positive component engaged approximately 300–500 ms after the NoGo stimulus, reflects conflict resolution and cognitive processing, including stimulus recognition, evaluation, and suppression of the actual motor response, representing the later stages of inhibitory control [13]. Potentials for both EEG components are more pronounced in the central frontal scalp region [14] and are commonly used as an indicator of response inhibition.

Many studies have shown that TSD has a negative effect on inhibitory control. At the behavioral level, Liu (2022) [8] found that one night of TSD significantly decreased individuals’ response inhibition and response execution. In addition, several studies have used event-related potential (ERP) to investigate the neurophysiological mechanisms underlying the effects of TSD on inhibitory control. For example, Qi (2010) [15] studied the effects of 43 h of TSD (43-h TSD) on inhibitory control using a GNG task and found that the amplitudes of NoGo-N2 and NoGo-P3 were significantly reduced compared to controls. Gosselin (2019) [16] performed auditory GNG tasks on 11 participants during 36-h TSD and found that NoGo-P3 amplitudes were significantly reduced after 24- and 36-h TSD. However, Liu (2015) [17] showed that there was no significant difference in NoGo-N2 amplitude after 72-h TSD compared to baseline. Jin et al., (2015) [18] asked participants to complete the visual GNG task after 12-, 24- and 36-h TSD. The results showed that the N2 latency was prolonged and the P3 amplitude decreased after 24-h TSD, but no significant change in N2 amplitude was found. In summary, TSD decreased P3 amplitude in inhibitory control tasks [16,19], but the effect of TSD on the N2 component remains controversial; some studies have found that N2 amplitude decreases and latency increases [15,20], while others have found no changes in N2 amplitude or latency [17,18,21]. These different results are presumably influenced by the duration of sleep deprivation, the type of inhibitory control task and the participants’ gender and age. Therefore, this study’s first aim was to measure inhibitory control function using a visual GNG task, to explore the effect of 36 h of TSD on inhibitory control in terms of time course using the ERP technique.

The brain is in a constant state of activity which persists (and can be recorded using EEG) even during sleep or when not engaged in any task. The brain regions that show increased synchronization of activity signals during resting states are called resting-state networks. The most studied resting-state network is the default mode network (DMN); it has the highest degree of activation in the resting state. The DMN, mainly including the posterior cingulate gyrus, medial prefrontal cortex, medial temporal lobe, and inferior parietal gyrus, plays a key role in the integration of cognitive processes [22]. Verweij (2014) [23] used high-density resting-state EEG to show that TSD strongly affects the functional connectivity (FC) of the prefrontal cortex. Previous studies have also used functional magnetic resonance imaging (fMRI) techniques with high spatial resolution to explore the effects of TSD on inhibitory control. Gujar et al., (2010) [24] showed that one night of TSD leads to imbalanced activation of anterior and posterior midline brain regions associated with the DMN. Yang et al., (2018) [25] analyzed the effect of TSD on the spontaneous activity of the brain using voxel-based FC density. The results showed that FC density of the posterior cingulate gyrus, precuneus, inferior parietal lobule, and dorsomedial prefrontal cortex decreased during mental fatigue, while FC density in the sensory integration and arousal regulation areas, including the postcentral gyrus, thalamus, and middle temporal gyrus, increased. These changes suggest that after TSD the brain may enhance perceptual activity and weaken advanced cognitive processing activities to achieve functional balance. However, more studies on DMN during TSD are needed.

To obtain more detailed frequency band information than is possible with fMRI, in the current study we used the resting-state FC analysis technique to further explore the effects of TSD on inhibitory control. Its high sampling rate allows us to extract various electrophysiological features in the frequency band of 0.1–40 Hz. The power of the α band is a good indicator of EEG vigilance [26], and has a major role in the process of closing eyes [27]. In addition, the α band is closely related to functions such as inhibition control and attention [28]. Boonstra (2005) [29] showed reduced alpha activity in the frontal cortex after 24-h TSD. However, the effect of TSD on alpha activity requires further exploration, so the present study focused specifically on changes in the alpha band (8–14 Hz) during TSD. The second purpose of this study was to explore the variations in resting-state brain network changes before and after TSD in the alpha band (8–14 Hz), using the resting-state functional connectivity technique, and to correlate the brain network connectivity changes with the changes in electroencephalogram components to explore the effect of TSD on inhibitory control, from the perspective of spatial characteristics.

In this study, 25 participants were directed to complete resting-state EEG acquisition and GNG tasks before and after 36-h TSD. ERP technology was used to explore the changes of amplitude and latency of EEG components NoGo-N2 and NoGo-P3, and to further explore FC changes in resting-state brain networks in the alpha band (8–14 Hz), and their correlation with inhibitory control function. The overall purpose was to reveal the neuroelectrophysiological mechanism of the effect of TSD on inhibitory control in the time course of cognitive processing and brain networks. The present study proposes two hypotheses: (1) 36 h of TSD can impair inhibitory control, as reflected by increased false alarms of NoGo stimuli in behavioral indicators, decreased amplitude, and prolonged latency of electrophysiological indicators NoGo-N2 and NoGo-P3 after TSD; and (2) functional connectivity between resting-state brain networks is reduced, especially in the DMN.

## 2. Materials and Methods

### 2.1. Participants

A total of 25 healthy, right-handed male participants aged 18–24 years (21.94 ± 1.73 years) with normal or corrected-to-normal visual acuity were recruited for this trial. Only male participants were recruited to avoid the effect of any potential gender differences. Participants were required not to have previously participated in relevant psychophysiological tests; not to have taken drugs recently; not to have a habit of smoking or drinking coffee, tea; no history of alcohol or drug abuse; and no history of mental illness. Before the formal trial, participants were required to complete the Pittsburgh Sleep Quality Index questionnaire (PSQI). The PSQI is a self-rated questionnaire that assesses the participant’s sleep quality during the most recent month. All participants scored less than 5, indicating good sleep quality [30]. The process and risks of sleep deprivation were completely explained to all participants, and written informed consent was obtained from them prior to the study’s initiation. The experiment was approved by the Ethics Committee of Beihang University. Appropriate payments were made to participants at the end of the experiment.

### 2.2. Experimental Tasks and Procedures

#### 2.2.1. Visual Go/NoGo Task

**Figure 1:**
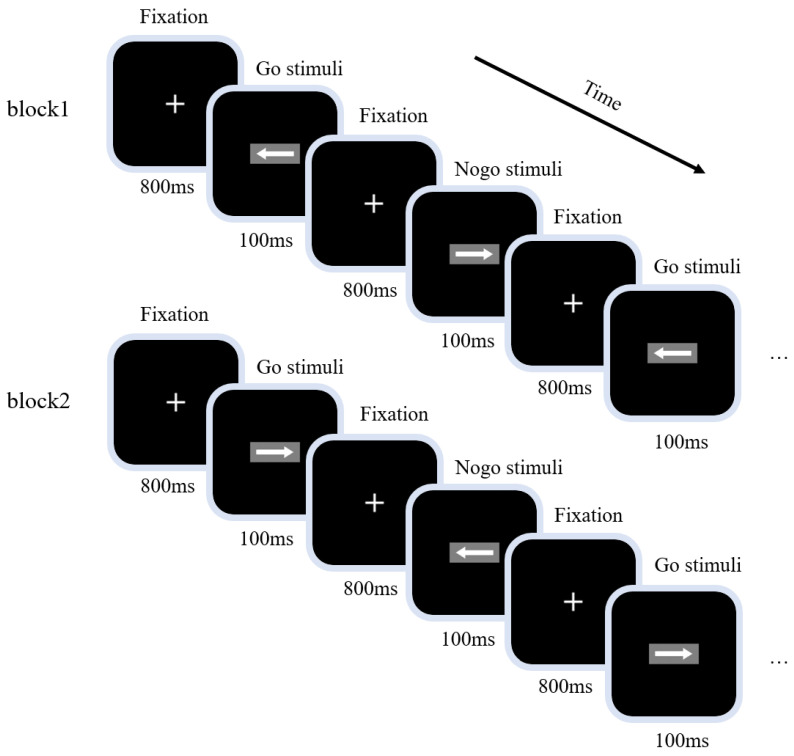
Visual Go/NoGo task diagram. The task was divided into two blocks, each containing 200 trials. Go and NoGo stimuli accounted for 2/3 and 1/3 of all stimuli, respectively; the presentation time of each stimulus was 100 ms and the interval between two adjacent stimuli was 800 ms.

The experiment used a visual GNG task to measure the inhibitory control function. The experimental procedure is shown in Figure 1; a 100 ms fixation point “+” was presented first, before Go or NoGo stimuli were presented at random (the stimulus signals were “left” and “right” arrows; size 2.0 cm × 0.5 cm, 1.5° wide and 0.4° high). Each stimulus was presented for 100 ms, with Go and NoGo stimuli accounting for 2/3 and 1/3 of all stimuli, respectively. The time interval between two adjacent stimuli was 800 ms. The task was divided into two blocks, each containing 200 trials. The first block directed participants to respond to the “left” arrow (Go stimulus) and not to the “right” arrow (NoGo stimulus); the instructions for the second block were the inverse. For the balance test, participants were required to respond to the “right” arrow (Go stimulus) and not to the “left” arrow (NoGo stimulus). The order of completion of the two blocks was balanced among participants; participants were told to respond as quickly and accurately as possible throughout the task.

#### 2.2.2. Experimental Procedures

**Figure 2:**
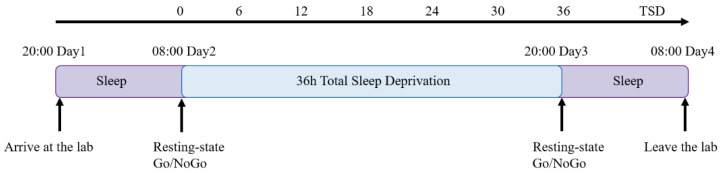
Flowchart showcasing the sleep deprivation experiment, with 60 h of experimental time. Participants entered the laboratory at 20:00 on the first night of the experiment and slept normally in the laboratory. The behavioral and electroencephalogram (EEG) data of the first resting-state and Go/NoGo task were collected at 08:00 on the second day (TSD: 0 h). On the third night, the behavioral and EEG data of the second resting-state and Go/NoGo task were collected after 35.5 ± 1.0 h of sleep deprivation (TSD: 36 h). Participants stayed in the laboratory for restorative sleep on the third night and left the laboratory at 8:00 a.m. on the fourth day.

Participants arrived at the laboratory at 20:00 the night before the start of the experiment, and the experimenter informed the participants in detail about the experimental procedure and the risks of TSD. Participants voluntarily signed the informed consent form, familiarized themselves with the laboratory environment, and practiced the visual GNG task. Participants slept in the laboratory on the first day and were guaranteed more than 8 h of sleep. The baseline measurement was performed at 08:00 a.m. (0-h TSD) on the second day. The three-minute closed-eye resting-state data acquisition and visual GNG task were completed, and EEG data were recorded. The whole test process was carried out in a shielded room with moderate sound insulation and lighting. On the third night of the experiment, after 35.5 ± 1.0 h of sleep deprivation (36-h TSD), the three-minute closed-eye resting-state data acquisition and visual GNG task were completed again. During the entire TSD period, participants were supervised by two experimenters and one physician to prevent them from sleeping. In each experiment, the participants’ sleep deprivation pre- and post-test data were collected and recorded by the same experimenter. We provided participants with three meals, including the necessary staples, vegetables, meat, and fruit, which was comparable to their regular food intake. Participants were not allowed to leave the lab during the experiment, were not allowed to engage in high-intensity physical activity, and were not allowed to eat food or beverages containing stimulants or caffeine. After the experiment, the experimenter rewarded and thanked the participants. At the same time, the participants were advised to leave after restorative sleep in the laboratory. We controlled the ambient temperature between 20 and 26 degrees and the relative humidity between 30% and 60% throughout the experiment, to provide a relatively constant temperature and humid environment for the participants. The experimental process is shown in Figure 2.

### 2.3. Data Collection

The distance between the participant and the monitor during the test was 75 cm, and the center of the participant’s visual field was level with the center of the monitor. The experimental program was written and presented using E-prime 2.0 professional software (Psychology Software Tools, Inc., Pittsburgh, PA, USA). The Neuroscan 32-channel electrode caps combined with a SynAmps2 amplifier (Compumedics Neuroscan, Charlotte, NC, USA) was used to collect EEG signals according to the 10–20 international EEG recording system. The participants’ vertical and horizontal EEG were recorded during the acquisition, and the bilateral mastoids were used as online reference electrodes. The EEG was sampled at a rate of 1000 Hz and the impedance of the electrodes was kept below 5 kΩ. Under both baseline and 36-h TSD conditions, participants completed resting-state and GNG tasks while recording EEG.

### 2.4. Data Analysis

#### 2.4.1. Behavioral Statistical Analysis

The behavioral data indicators included reaction times and hit rates for Go stimuli and false alarms of NoGo stimuli. False alarms of NoGo stimuli indicate the creation of a keypress response, when there should not be any keypress response to a NoGo stimulus. SPSS v26.0 (IBM SPSS Statistics, Armonk, NY, USA) software was used to perform paired sample *t*-tests on these indicators.

#### 2.4.2. Data Preprocessing and ERP Statistical Analysis

EEG preprocessing and ERP analysis were performed using EEGLAB R2018b (MathWorks Inc., Natrick, MA, USA) with EEGLAB v21.1.0 [31] and ERPLAB v8.10 [32]. Preprocessing began by checking for abnormal EEG data, followed by average re-reference and band-pass filtering from 0.1 to 40 Hz. Next, independent component analysis (ICA) was used to remove artifacts such as eye-electricity and electromyography [33]. The sampling rate was reduced to 250 Hz. Baseline correction was performed from 200 ms before stimulus presentation to stimulus presentation, and segmentation was performed from −200 ms to 800 ms. Segments with amplitudes exceeding ±100 μV were removed. After completing the preprocessing, ERPLAB v8.10 was used to average the correct responses.

A combination of previous studies [11,34,35] and the total waveform and topographic maps of this study helped determine the chosen time windows of N2 and P3 to be 200–300 ms and 350–450 ms, respectively. The amplitude and latency of the N2 (200–300 ms) and P3 (350–450 ms) components were calculated by selecting the average of the Fz, F3, F4, FCz, FC3, and FC4 electrodes. The amplitude and latency of N2 and P3 components were quantified as the maximum amplitude and corresponding time points between 200 and 300 ms, and 350 and 450 ms.

#### 2.4.3. EEG Source Connectivity Analysis

Resting-state data from all 25 participants were preprocessed using EEGLAB v21.1.0. EEG data were first browsed to check for abnormal data and resting-state data from 1 participant were removed due to excessive head movement and EEG artifacts, and data from 24 participants were included in the analysis. Next, the useless electrodes are deleted for average re-reference and 0.1–40 Hz band-pass filtering. The sampling rate was reduced to 250 Hz. The data were divided into 2 s segments, based on the previous literature on resting-state EEG data analysis [36,37,38,39].

Using FieldTrip [40] for source localization and FC analysis, we chose the alpha band of interest (8–14 Hz) for analysis. First, the “ft_freqanalysis” function was used to conduct a fast Fourier transform based on the Hanning window, then the “ft_sourceanalysis” function was used for source localization. The head model used the standard custom boundary element method head volume conduction model in FieldTrip [41] and then used partial canonical correlation/coherence as the beamformer method to calculate the spatial filter [42]. Our input was the EEG signal of the remaining 30 electrodes and the output was the reconstructed time course of 4050 dipoles, constructed for neural activity throughout the cerebral cortex. We used an anatomical automatic labeling 90 template to divide the brain into 90 brain regions [43], and then the “ft_connectivityanalysis” function to calculate the phase locking value (PLV) of 90 brain regions; finally, we obtained a 24 × 90 × 90 FC matrix.

Commonly used FC metrics for EEG include coherence, Pearson correlation coefficient, phase lag index, and PLV. Because EEG recordings are susceptible to volume conduction effects, they are susceptible to interference from sources at other sites when constructing FC, and therefore pose problems for determining FC between signals using traditional algorithms such as coherence and synchronization likelihood [44]. Among these metrics, PLV reflects the phase-synchronous relationship between two signals, has the advantage of being less computationally intensive and more applicable to non-stationary signals, and is commonly used in functional brain connectivity analysis [45]. In view of this, PLV was selected as an important indicator of phase synchronization in this study, by which the degree of phase change between two signals is measured.

NBS software [46] was used to implement the nonparametric network-based statistic method, which is commonly used in network analysis. In this study, NBS v1.2 was used to perform paired sample *t*-tests for the difference between the 24 × 90 × 90 FC matrix in both baseline and 36-h TSD conditions with a significance level of 0.05; the nonparametric network-based statistic method was chosen for multiple correction.

#### 2.4.4. Correlation Analysis

To further explore the relationship between FC changes and EEG component amplitude changes, Pearson correlation analysis was performed using SPSS v26.0 for significantly different PLV changes (TSD–baseline) and amplitude changes of NoGo-N2 and NoGo-P3 (TSD–baseline).

## 3. Results

### 3.1. Behavioral Performance

**Figure 3:**
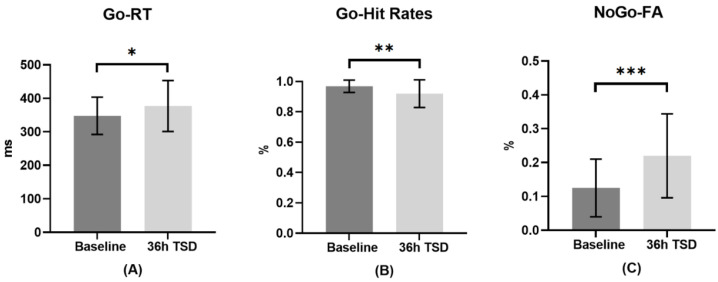
The differences in reaction times (RT) and hit rates in Go trials and false alarms (FA) in NoGo trials at baseline and 36-h total sleep deprivation (TSD). (**A**): Go-RTs: Go stimulus reaction times at baseline and 36-h TSD; (**B**): Go-Hit Rates: Go stimulus hit rates at baseline and 36-h TSD; (**C**): NoGo-FA: NoGo stimulation false alarms at baseline and 36-h TSD. Error bars represent standard deviation. *: *p* < 0.05, **: *p* < 0.01, ***: *p* < 0.001.

The means, standard deviations and *t*-test results of the hit rates and reaction times of Go stimuli and the false alarms of NoGo stimuli at baseline and 36-h TSD are shown in Table 1. The effects of sleep conditions (baseline and 36-h TSD) on behavioral indicators are shown in Figure 3. The results of paired sample *t*-tests showed that compared with baseline, reaction times to Go stimuli after 36-h TSD significantly increased (*t* = −2.412, *p* < 0.05, Cohen’s d = 0.482), hit rates of Go stimuli significantly decreased (*t* = 3.158, *p* < 0.01, Cohen’s d = 0.632), and false alarms for NoGo stimuli significantly increased (*t* = −4.187, *p* < 0.001, Cohen’s d = 0.837).

### 3.2. ERP Results

**Figure 4:**
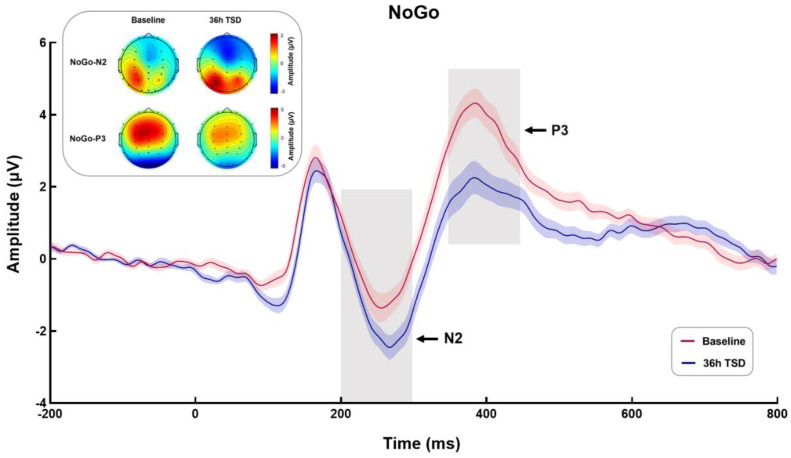
Event-related potential (ERP) and topographic maps of NoGo-N2 (200–300 ms) and NoGo-P3 (350–450 ms) at baseline and 36-h total sleep deprivation (TSD). ERP was calculated by averaging the data at Fz, F3, F4, FCz, FC3, and FC4 electrodes. The red and blue curves represent the ERP for the baseline and 36-h TSD conditions, respectively; the shaded area around each line represents the standard deviation of the signal divided by the square root of the number of channels.

The means and standard deviations of NoGo-N2 and NoGo-P3 amplitudes and latencies during TSD are shown in Table 2. The waveforms of NoGo stimulation and the topographic maps of NoGo-N2 (200–300 ms) and NoGo-P3 (350–450 ms) are shown in Figure 4.

#### 3.2.1. NoGo-N2

Paired sample *t*-tests were performed on the amplitude and latency of NoGo-N2. The results showed that compared with the baseline, NoGo-N2 showed a larger negative peak and a longer latency after 36-h TSD (*t* = 4.850, *p* < 0.001, Cohen’s d = 0.970; *t* = −3.178, *p* < 0.01, Cohen’s d = 0.636).

#### 3.2.2. NoGo-P3

Paired sample *t*-tests showed a significant decrease in NoGo-P3 amplitude and prolonged latency after 36-h TSD compared to baseline (*t* = 5.104, *p* < 0.001, Cohen’s d = 1.021; *t* = −2.382, *p* < 0.05, Cohen’s d = 0.476).

### 3.3. Functional Connectivity Results

**Figure 5:**
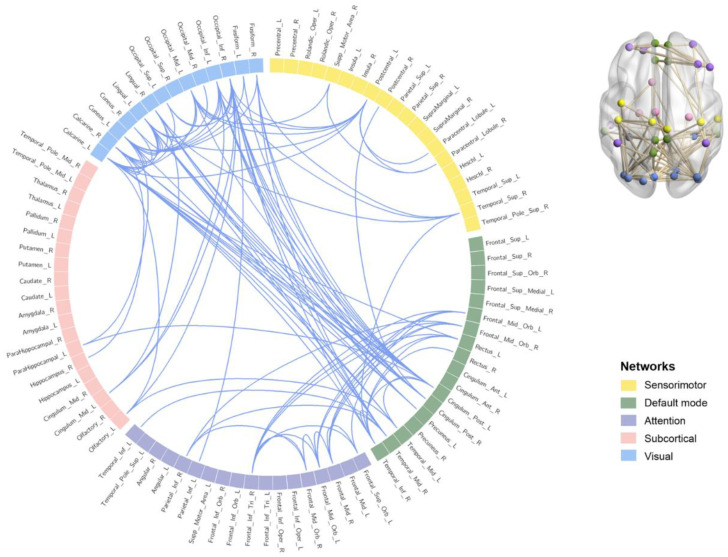
Whole brain functional connectivity results. The squares represent 90 brain regions. The yellow squares represent sensorimotor networks, the green squares represent default mode networks, the purple squares represent attention networks, the pink squares represent subcortical networks, and the blue squares represent visual networks. The connection is a significant functional connection.

FC between 90 brain regions was tested for significance. The results showed that 101 brain regions were significantly related to FC in the high α band (11–14 Hz) (*t* = 2.500, *p* = 0.030). No significant FC was found in the 8–11 Hz (*p* > 0.05). The results are shown in Figure 5 (See Supplementary Materials for details). Brainnet Viewer v1.63 [47] and Circos v0.69–6 [48] were used to visualize the results.

### 3.4. Correlation Results

The correlation results showed that ∆N2 amplitude (TSD–baseline) was significantly negatively correlated with ∆PLV (TSD–baseline) of the right orbital middle frontal gyrus and the right deltoid inferior frontal gyrus (IFGtriang.R; *r* = −0.409, *p* = 0.047), IFGtriang.R and the left gyrus rectus (*r* = −0.434, *p* = 0.034), the left posterior cingulate gyrus (PCG.L) and the left parahippocampal gyrus (*r* = −0.503, *p* = 0.012), left middle occipital gyrus (MOG.L) and right inferior occipital gyrus (IOG.R) (*r* = −0.483, *p* = 0.017), PCG.L and right fusiform gyrus (FFG.R) (*r* = −0.471, *p* = 0.020), left cuneus (CUN.L) and FFG.R (*r* = −0.458, *p* = 0.024), PCG.L and right postcentral gyrus (*r* = −0.436, *p* = 0.033), PCG.L and right inferior temporal gyrus (*r* = −0.498, *p* = 0.013).

**Figure 6:**
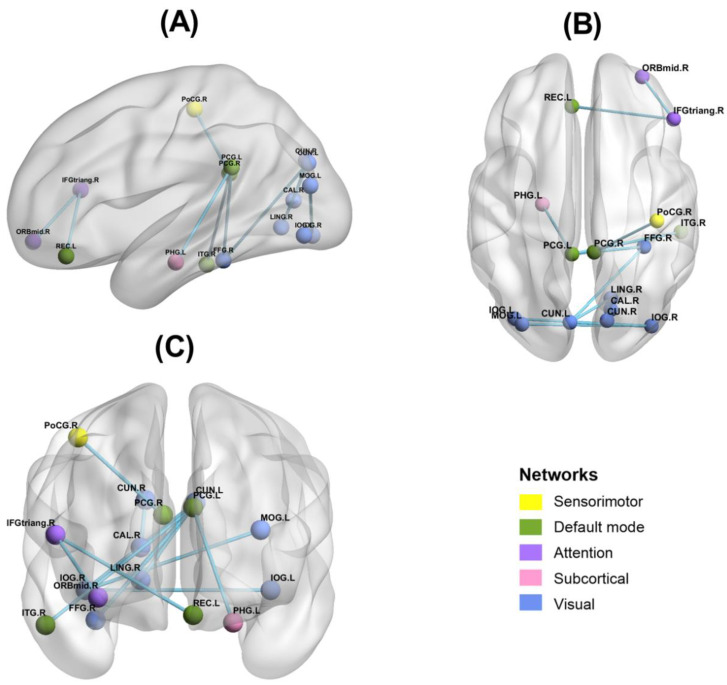
Results of functional connectivity of brain regions with significant correlation to N2 and P3 amplitude changes. (**A**): Single view: Sagittal; (**B**): Single view: Axial; (**C**): Single view: Coronal. Yellow is sensorimotor network, green is default mode network, purple is attention network, pink is subcortical network, and blue is visual network. ORBmid.R: right orbital middle frontal gyrus; IFGtriang.R: right triangle inferior frontal gyrus; REC.L: left gyrus rectus; PCG.L: left posterior cingulate gyrus; PHG.L: left parahippocampal gyrus; MOG.L: left middle occipital gyrus; IOG.R: right inferior occipital gyrus; FFG.R: right fusiform gyrus; CUN.L: left cuneus; PoCG.R: right postcentral gyrus; ITG.R: right inferior temporal gyrus; CUN.R: right cuneus; CAL.R: right calcarine fissure surrounding cortex; LING.R: right lingual gyrus; IOG.L: left inferior occipital gyrus.

**Figure 7:**
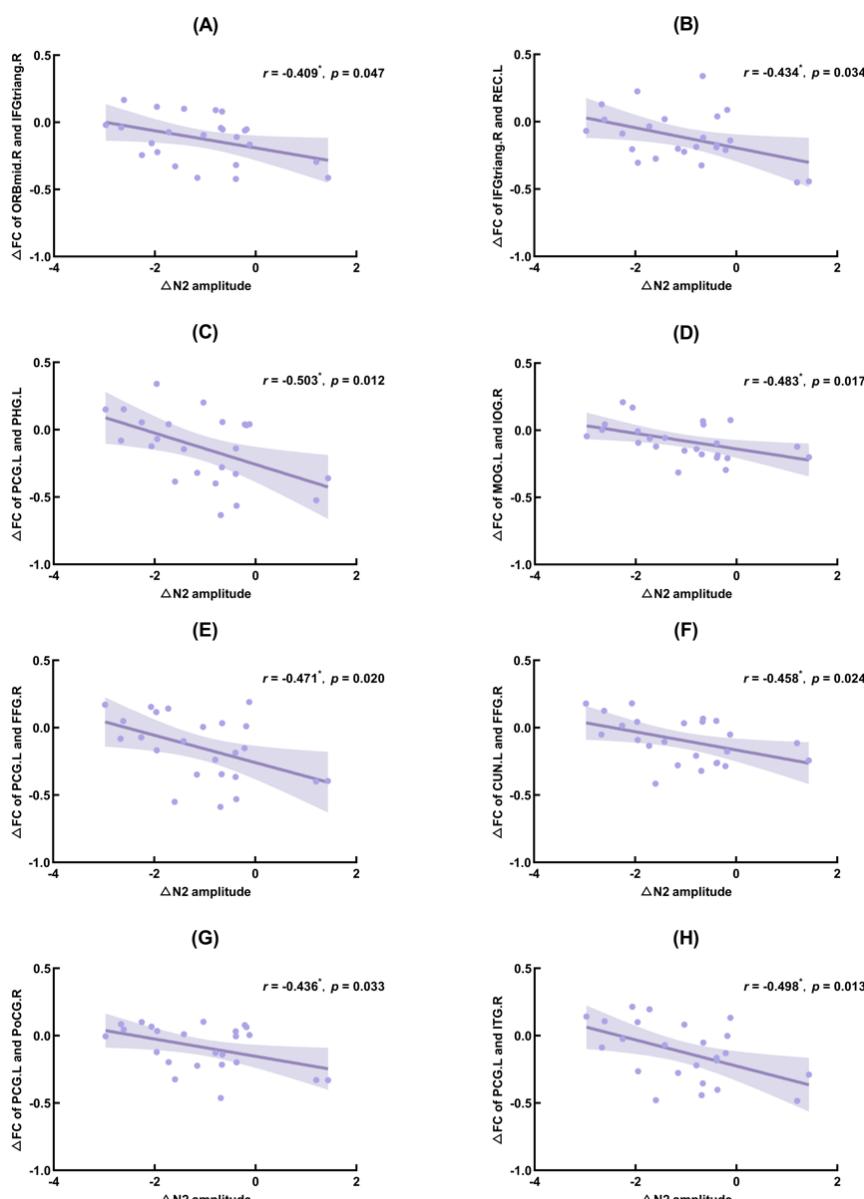
Significant correlation between functional connectivity (FC) changes and N2 amplitude changes. ORBmid.R: right orbital middle frontal gyrus; IFGtriang.R: right triangle inferior frontal gyrus; REC.L: left gyrus rectus; PCG.L: left posterior cingulate gyrus; PHG.L: left parahippocampal gyrus; MOG.L: left middle occipital gyrus; IOG.R: right inferior occipital gyrus; FFG.R: right fusiform gyrus; CUN.L: left cuneus; PoCG.R: right postcentral gyrus; ITG.R: right inferior temporal gyrus. (**A**): Correlation between ∆FC of ORBmid.R and IFGtriang.R and ∆N2 amplitude; (**B**): Correlation between ∆FC of IFGtriang.R and REC.L and ∆N2 amplitude; (**C**): Correlation between ∆FC of PCG.L and PHG.L and ∆N2 amplitude; (**D**): Correlation between ∆FC of MOG.L and IOG.R and ∆N2 amplitude; (**E**): Correlation between ∆FC of PCG.L and FFG.R and ∆N2 amplitude; (**F**): Correlation between ∆FC of CUN.L and FFG.R and ∆N2 amplitude; (**G**): Correlation between ∆FC of PCG.L and PoCG.R and ∆N2 amplitude; (**H**): Correlation between ∆FC of PCG.L and ITG.R and ∆N2 amplitude. *: *p* < 0.05.

**Figure 8:**
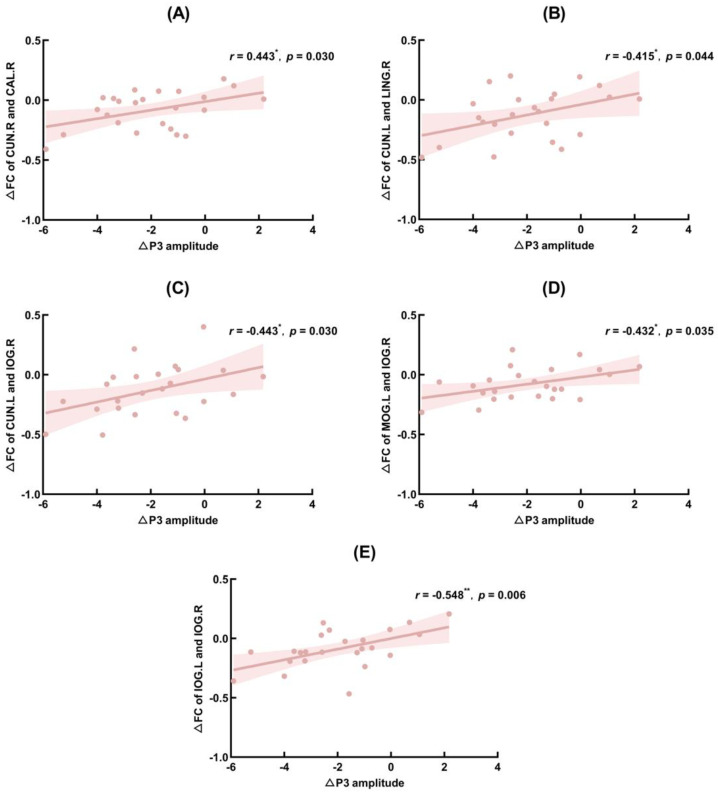
Significant correlation between functional connectivity (FC) changes and P3 amplitude changes. MOG.L: left middle occipital gyrus; IOG.R: right inferior occipital gyrus; CUN.L: left cuneus; CUN.R: right cuneus; CAL.R: right calcarine fissure surrounding cortex; LING.R: right lingual gyrus; IOG.L: left inferior occipital gyrus. (**A**): Correlation between ∆FC of CUN.R and CAL.R and ∆P3 amplitude; (**B**): Correlation between ∆FC of CUN.L and LING.R and ∆P3 amplitude; (**C**): Correlation between ∆FC of CUN.L and IOG.R and ∆P3 amplitude; (**D**): Correlation between ∆FC of MOG.L and IOG.R and ∆P3 amplitude; (**E**): Correlation between ∆FC of IOG.L and IOG.R and ∆P3 amplitude. *: *p* < 0.05; **: *p* < 0.01.

∆P3 amplitude (TSD–baseline) was significantly positively correlated with ∆PLV (TSD–baseline) of the right cuneus and right calcarine fissure and surrounding cortex (*r* = 0.443, *p* = 0.030), CUN.L and right lingual gyrus (*r* = 0.415, *p* = 0.044), CUN.L and IOG.R (*r* = 0.443, *p* = 0.003), MOG.L and IOG.R (*r* = 0.432, *p* = 0.035), and left inferior occipital gyrus and IOG.R (*r* = 0.548, *p* = 0.006). The FC of brain regions that correlated significantly with ∆N2 and ∆P3 amplitudes are shown in Figure 6, Figure 7 and Figure 8, showing the results of significant correlation between ∆FC and ∆N2 amplitudes, and ∆FC and ∆P3 amplitudes, respectively.

## 4. Discussion

In this study, the visual GNG task was used to explore the effect of 36-h TSD on inhibitory control and its neuroelectrophysiological mechanism through ERP and FC analysis methods. These effects were manifested as behavioral indicators (reaction times and hit rates for Go stimuli, false alarms for NoGo stimuli), amplitude and latency of NoGo-N2 and NoGo-P3, and changes in the FC of brain regions. Previous studies have shown that biological rhythms can have important effects on cognitive function [49]; we chose to avoid biological lows and measured participants’ behavioral indicators and EEG data at 08:00 and 20:00. The results showed that, participants had impaired inhibitory control after 36-h TSD, with a significant increase in response times to Go stimuli and false alarms for NoGo stimuli, and a significant decrease in hit rates for Go stimuli. The ERP and FC results showed that the amplitudes of NoGo-N2 and NoGo-P3 separated, and the FC of the DMN and the visual network decreased significantly after TSD.

Behavioral results suggest that 36-h TSD resulted in participants becoming more impulsive and experiencing greater difficulty inhibiting inappropriate responses due to lack of sleep [50]; their inhibitory control was impaired. In this study, ERP technology was used to further explore the neuroelectrophysiological mechanism of the effect of TSD on inhibitory control. The results with respect to NoGo-P3 were consistent with previous studies [15,18]. Compared with the baseline, participants’ NoGo-P3 amplitude after 36-h TSD was significantly reduced, the latency was prolonged, and the energy in the central frontal region was reduced (Figure 4). A study by Gosselin (2019) [16] using an auditory GNG task found a significant reduction in NoGo-P3 amplitude after 24- and 36-h TSD. In addition, the results of a meta-analysis showed that sleep restriction can have a more significant negative effect on behavioral inhibition [51]. Most researchers believe that NoGo-P3 represents the time required for the inhibition of actual motor responses and the classification and evaluation of stimuli. It may be related to the completion of the psychological process [52], and it is usually used as an indicator of response inhibition. In our study, compared with the baseline, the significant decrease of NoGo-P3 amplitude and the significant prolongation of latency after 36-h TSD reflected that participants’ decision-making process gradually became more uncertain during TSD, and the late task-related advanced cognitive processing process was damaged to a certain extent after TSD; that is, TSD significantly reduced participants’ ability to classify and evaluate stimuli.

While the results of NoGo-P3 were similar to previous studies, the situation is more complicated with respect to NoGo-N2, about which previous studies have found mixed results: some found a decrease in NoGo-N2 amplitude and prolonged latency after TSD [15,20], while others found no changes [17,18]. Fueggle (2018) [53] conducted a study of 60 young adults over 7 days of sleep monitoring and did not find significant differences in N2 amplitude and latency between the longer and shorter sleep groups. A study by Kusztor (2019) [21] used the stop-signal paradigm to separate the different processes of task execution and found that TSD led to impaired top-down cognitive control in the late stages, but had insignificant effects on early automatic control. Yang et al., (2018) [25] showed that FC in the posterior cingulate gyrus, precuneus, inferior parietal lobule, and dorsomedial prefrontal cortex was reduced during fatigue, whereas FC in sensory integration and arousal regulation areas was enhanced; this may represent the possibility that the brain enhances perceptual activity and attenuates higher cognitive processing activity to achieve functional balance after TSD. NoGo-N2 typically reflects early monitoring of conflict and the allocation of attentional resources, indicating pre-motor inhibition processes [11]. The present study found a significant increase in the negative amplitude of NoGo-N2 after 36-h TSD, with a prolonged latency and increased energy in the central frontal region (Figure 4). This result is not entirely consistent with our hypothesis. The alertness hypothesis suggests that only individuals with a certain level of alertness are able to excel in subsequent tasks [54]. We speculate that the significant increase in the negative amplitude may represent an increase in the degree of sleepiness of the participants after 36 h of TSD compared to the baseline. Therefore, more sustained attention and vigilance may be required to focus on the current task, so as to ensure smooth progress, and the brain also enhances perceptual activity. The increased energy in the center of the forehead also suggests that participants invested more attention and cognitive resources in completing tasks after TSD.

This study found that FC between brain networks was significantly reduced after 36-h TSD in the high alpha band; FC between the DMN and the visual network was especially greatly affected after TSD. Further, this study did not identify significant FC changes in the low alpha band. Some studies have suggested that high alpha activity may indicate inhibited task-irrelevant brain areas or an overall reduction in brain activity [55,56]. We speculate the reason for this result to be the reduced FC between resting-state brain networks, primarily in the high alpha band after TSD. Some studies have shown that FC within the DMN is significantly reduced and compromised after TSD [24]. Kaufmann (2015) [57] found that TSD dynamically altered the connectivity of the dorsal attention network, DMN, and the hippocampal network. This study found that FC within the visual network and between the DMN and the visual network was significantly reduced after TSD, which may indicate that after 36-h TSD, the early automatic attention and alertness of the participants were impaired to some extent and the spontaneous activity of the DMN brain region was impaired [58].

In this study, the significant FC results were correlated with the amplitude changes of N2. The results showed that the amplitude changes of N2 were significantly negatively correlated with the FC changes of posterior cingulate gyrus and parahippocampal gyrus, fusiform gyrus, postcentral gyrus, inferior temporal gyrus, inferior frontal gyrus and middle frontal gyrus, rectus gyrus, middle occipital gyrus and inferior occipital gyrus, cuneus, and fusiform gyrus. In the DMN, the cingulate cortex is the backbone of the brain network involved in cognitive processes such as sleep and executive control [59,60,61]. In addition, the posterior cingulate gyrus is widely considered to be the central node of the network [62] and has particularly strong FC between DMN regions. A study by Coito (2019) [63] suggests that the posterior cingulate gyrus is the main driver of spontaneous brain activity. Previous studies have shown that the hippocampus contributes to conflict resolution and control of behavioral inhibition and is specifically sensitive to sleep deprivation [64]; the right inferior frontal gyrus also plays a key role in cognitive control and is specifically involved in inhibiting [65,66,67]; the inferior temporal gyrus is also closely related to visual information processing and is responsible for the early processing of a wide range of perceptual information; and the fusiform gyrus is positively activated in cognitive behaviors such as object recognition, facial recognition, limb and character recognition. The postcentral cortex is the primary somatosensory area of the brain, which is responsible for processing of within-the-body perceptual information. The occipital visual cortex is mainly responsible for visual information collection, processing and conversion. Bosch’s (2013) [68] fMRI study showed that TSD reduced the resting-state FC between the posterior cingulate gyrus and bilateral anterior cingulate cortex in patients with depression. Dai (2015) [58] found that, compared with normal sleep, the resting-state FC of the right inferior parietal lobule and the left precuneus and posterior cingulate cortex decreased after TSD, and the resting-state FC of the left fusiform gyrus and inferior temporal gyrus increased. Therefore, we speculate that the decrease in FC may represent a decrease in visual information processing and processing functions after 36-h TSD, and the impairment of the DMN. There was a significant negative correlation between the amplitude change of N2 and the change of FC. That is to say, as sleep deprivation advanced, the N2 amplitude gradually increased while the FC changes gradually decreased, which may indicate that although the connection between the visual and default mode networks was damaged after sleep deprivation, the brain may attempt to mitigate this damage to provide participants with more attention to and recognition of task information, so as to ensure that participants can carry out tasks smoothly and perform appropriately.

In addition, this study also analyzed the correlation between significant FC results and P3 amplitude changes. The results showed that the amplitude changes of P3 were significantly positively correlated with the FC changes of the cuneus and calcarine cortex, lingual gyrus, suboccipital gyrus, middle occipital gyrus, and suboccipital gyrus. The cuneus is an important brain region for higher visual cortex, visual information processing, spatial orientation, somatosensory integration, and self-awareness [69]. The cortex around the talus fissure, the lingual gyrus and the inferior occipital gyrus are all located in the occipital lobe, of which the function is to perceive and process visual information and participate in the complex visual perception process. Kong (2011) [70] showed that TSD reduced visual processing capacity and activation of the cingulate gyrus region. In addition, Zhao (2018) [71] found that brain activation in visual-related areas (occipital cortex, lingual gyrus, and cingulate gyrus) was reduced after TSD. These findings suggest that TSD has the potential to impair visual processing abilities. The present study found that FC between the cuneate and occipital regions (calcarine fissure and surrounding cortex, lingual gyrus, and inferior occipital gyrus) was significantly reduced after sleep deprivation and was significantly and positively correlated with P3 amplitude change. In other words, during sleep deprivation, the amplitude of P3 decreased gradually, and the functional connection between the cuneus and occipital lobe also decreased, which may represent the impairment of participants’ advanced visual information processing ability, which in turn reduced their ability to classify and evaluate stimuli, resulting in the decrease of individuals’ inhibitory control ability when completing tasks.

Although our study has made contributions to the literature, it presents some limitations. First, no female participants were recruited to mitigate any effect of sex differences, so there may be limitations in generalizing the findings to different populations. Second, circadian factors have also been shown to affect FC outcomes [72], so the results may include the effect of circadian rhythms. Third, the current study examined only the alpha band’s resting-state EEG changes, while changes in other frequency bands were not taken into account. Future studies could incorporate fMRI techniques to provide more insight into the neurophysiological mechanisms underlying the effects of sleep deprivation on inhibitory control.

## 5. Conclusions

This study used the visual GNG task to explore the effect of 36 h sleep deprivation on inhibitory control from the perspective of neuroelectrophysiology and brain networks. Participants may have invested more attention and cognitive resources after 36-h TSD, while advanced cognitive processing, the DMN, and visual information processing were impaired to some extent, explaining the observed changes in ERP components.

## Figures and Tables

**Table 1:** Means and standard deviations of reaction times (RT) and hit rates in Go trials and false alarms (FA) in NoGo trials at baseline and 36-h total sleep deprivation (TSD) and *t*-test results.

Behavioral Variables	Baseline	36 h-TSD	*t*	*p*
Go-RT (ms)	347.83 ± 55.55	377.13 ± 75.95	−2.412 *	0.0240
Go-Hit Rates (%)	0.968 ± 0.041	0.919 ± 0.091	3.158 **	0.0040
NoGo-FA (%)	0.125 ± 0.085	0.220 ± 0.124	−4.187 ***	0.0003

*: *p* < 0.05; **: *p* < 0.01; ***: *p* < 0.001.

**Table 2:** Amplitude and latency (mean ± standard deviation) of NoGo-N2 and NoGo-P3 at baseline and 36-h total sleep deprivation (TSD) and *t*-test results.

		Baseline	36 h-TSD	*t*	*p*
NoGo-N2	Amplitude (μV)	−2.03 ± 2.20	−3.15 ± 1.76	4.85 ***	<0.001
	Latency (ms)	257.28 ± 22.19	267.76 ± 21.72	−3.178 **	0.004
NoGo-P3	Amplitude (μV)	4.80 ± 2.25	2.84 ± 2.62	5.104 ***	<0.001
	Latency (ms)	386.48 ± 21.32	398.96 ± 27.10	−2.382 *	0.025

*: *p* < 0.05; **: *p* < 0.01; ***: *p* < 0.001.

## Data Availability

The datasets generated for this study are available on request to corresponding authors.

## Supplementary Materials

The following supporting information can be downloaded at: https://www.mdpi.com/article/10.3390/ijerph20054663/s1, Table S1: 101 pairs of brain regions with significant functional connectivity.

## References

[1] Reynolds A.C., Banks S. (2010). Total sleep deprivation, chronic sleep restriction and sleep disruption. Prog. Brain Res..

[2] Dubal S., Jouvent R. (2004). Time-on-task effect in trait anhedonia. Eur. Psychiatry.

[3] Tucker A.M., Basner R.C., Stern Y., Rakitin B.C. (2009). The variable response-stimulus interval effect and sleep deprivation: An unexplored aspect of psychomotor vigilance task performance. Sleep.

[4] Williamson A.M., Feyer A.M. (2000). Moderate sleep deprivation produces impairments in cog-nitive and motor performance equivalent to legally prescribed levels of alcohol intoxication. Occup. Environ. Med..

[5] Gujar N., Yoo S.S., Hu P., Walker M.P. (2011). Sleep deprivation amplifies reactivity of brain reward networks, biasing the appraisal of positive emotional experiences. J. Neurosci..

[6] Telzer E.H., Fuligni A.J., Lieberman M.D., Galván A. (2013). The effects of poor quality sleep on brain function and risk taking in adolescence. NeuroImage.

[7] Gibbings A., Ray L.B., Berberian N., Nguyen T., Shahidi Zandi A., Owen A.M., Comeau F., Fogel S.M. (2021). EEG and behavioural correlates of mild sleep deprivation and vigilance. Clin. Neurophysiol..

[8] Liu S., Zhang R. (2022). Aerobic Exercise Alleviates the Impairment of Cognitive Control Ability Induced by Sleep Deprivation in College Students: Research Based on Go/NoGo Task. Front. Psychol..

[9] Diamond A. (2013). Executive functions. Annu. Rev. Psychol..

[10] Smith J.L., Johnstone S.J., Barry R.J. (2008). Movement-related potentials in the Go/NoGo task: The P3 reflects both cognitive and motor inhibition. Clin. Neurophysiol..

[11] Yuan J., Meng X., Yang J., Yao G., Hu L., Yuan H. (2012). The valence strength of unpleasant emotion modulates brain processing of behavioral inhibitory control: Neural correlates. Biol. Psychol..

[12] Mecklinger A., Parra M., Waldhauser G.T. (2009). ERP correlates of intentional forgetting. Brain Res..

[13] Khedr E.M., El Fetoh N.A., Gamal R.M., Elzohri M.H., Azoz N., Furst D.E. (2020). Evaluation of cognitive function in systemic sclerosis patients: A pilot study. Clin. Rheumatol..

[14] Wessel J.R. (2018). Prepotent motor activity and inhibitory control demands in different variants of the go/no-go paradigm. Psychophysiology.

[15] Qi J.L., Shao Y.C., Miao D., Fan M., Bi G.H., Yang Z. (2010). The effects of 43 hours of sleep deprivation on executive control functions: Event-related potentials in a visual go/no go task. Soc. Behav. Personal..

[16] Gosselin A., De Koninck J., Campbell K.B. (2019). Disentangling specific inhibitory versus general decision-making processes during sleep deprivation using a Go/NoGo ERP paradigm. Int. J. Psychophysiol..

[17] Liu Q., Zhou R., Liu L., Zhao X. (2015). Kuster deprivation on male astronauts’ executive functions and emotion. Compr. Psychiatry.

[18] Jin X., Ye E., Qi J., Wang L., Lei Y., Chen P., Mi G., Zou F., Shao Y., Yang Z. (2015). Recovery Sleep Reverses Impaired Response Inhibition due to Sleep Restriction: Evidence from a Visual Event Related Potentials Study. PLoS ONE.

[19] Boonstra T.W., Stins J.F., Daffertshofer A., Beek P.J. (2007). Effects of sleep deprivation on neural functioning: An integrative review. Cell. Mol. Life Sci..

[20] Renn R.P., Cote K.A. (2013). Performance monitoring following total sleep deprivation: Effects of task type and error rate. Int. J. Psychophysiol..

[21] Kusztor A., Raud L., Juel B.E., Nilsen A.S., Storm J.F., Huster R.J. (2019). Sleep deprivation differentially affects subcomponents of cognitive control. Sleep.

[22] Menon V. (2011). Large-scale brain networks and psychopathology: A unifying triple network model. Trends Cogn. Sci..

[23] Verweij I.M., Romeijn N., Smit D.J., Piantoni G., Van Someren E.J., van der Werf Y.D. (2014). Sleep deprivation leads to a loss of functional connectivity in frontal brain regions. BMC Neurosci..

[24] Gujar N., Yoo S.S., Hu P., Walker M.P. (2010). The unrested resting brain: Sleep deprivation al-ters activity within the default-mode network. J. Cogn. Neurosci..

[25] Yang L., Lei Y., Wang L., Chen P., Cheng S., Chen S., Sun J., Li Y., Wang Y., Hu W. (2018). Abnormal functional connectivity density in sleep-deprived subjects. Brain Imaging Behav..

[26] Olbrich S., Mulert C., Karch S., Trenner M., Leicht G., Pogarell O., Hegerl U. (2009). EEG-vigilance and BOLD effect during simultaneous EEG/fMRI measurement. NeuroImage.

[27] Larson-Prior L.J., Power J.D., Vincent J.L., Nolan T.S., Coalson R.S., Zempel J., Snyder A.Z., Schlaggar B.L., Raichle M.E., Petersen S.E. (2011). Modulation of the brain’s functional net-work architecture in the transition from wake to sleep. Prog. Brain Res..

[28] Yamagishi N., Callan D.E., Goda N., Anderson S.J., Yoshida Y., Kawato M. (2003). Attentional modulation of oscillatory activity in human visual cortex. NeuroImage.

[29] Boonstra T.W., Daffertshofer A., Beek P.J. (2005). Effects of sleep deprivation on event-related fields and alpha activity during rhythmic force production. Neurosci. Lett..

[30] Buysse D.J., Reynolds C.F., Monk T.H., Berman S.R., Kupfer D.J. (1989). The Pittsburgh Sleep Quality Index: A new instrument for psychiatric practice and research. Psychiatry Res..

[31] Delorme A., Makeig S. (2004). EEGLAB: An open source toolbox for analysis of single-trial EEG dynamics including independent component analysis. J. Neurosci. Methods.

[32] Lopez-Calderon J., Luck S.J. (2014). ERPLAB: An open-source toolbox for the analysis of event-related potentials. Front. Hum. Neurosci..

[33] Jung T.P., Makeig S., Humphries C., Lee T.W., McKeown M.J., Iragui V., Sejnowski T.J. (2000). Removing electroencephalographic artifacts by blind source separation. Psychophysiology.

[34] Baumeister S., Hohmann S., Wolf I., Plichta M.M., Rechtsteiner S., Zangl M., Ruf M., Holz N., Boecker R., Meyer-Lindenberg A. (2014). Sequential inhibitory control processes assessed through simultaneous EEG-fMRI. NeuroImage.

[35] Schmiedt-Fehr C., Basar-Eroglu C. (2011). Event-related delta and theta brain oscillations reflect age-related changes in both a general and a specific neuronal inhibitory mechanism. Clin. Neurophysiol..

[36] Hanslmayr S., Backes H., Straub S., Popov T., Langguth B., Hajak G., Bäuml K.H., Landgrebe M. (2013). Enhanced resting-state oscillations in schizophrenia are associated with decreased synchronization during inattentional blindness. Hum. Brain Mapp..

[37] Shephard E., Tye C., Ashwood K.L., Azadi B., Johnson M.H., Charman T., Asherson P., McLoughlin G., Bolton P.F. (2019). Oscillatory neural networks underlying resting-state, attentional control and social cognition task conditions in children with ASD, ADHD and ASD+ADHD. Cortex.

[38] Xie W., Toll R.T., Nelson C.A. (2022). EEG functional connectivity analysis in the source space. Dev. Cogn. Neurosci..

[39] Schorr B., Schlee W., Arndt M., Bender A. (2016). Coherence in resting-state EEG as a predictor for the recovery from unresponsive wakefulness syndrome. J. Neurol..

[40] Oostenveld R., Fries P., Maris E., Schoffelen J.M. (2011). FieldTrip: Open source software for advanced analysis of MEG, EEG, and invasive electrophysiological data. Comput. Intell. Neurosci..

[41] Oostenveld R., Stegeman D.F., Praamstra P., van Oosterom A. (2003). Brain symmetry and topo-graphic analysis of lateralized event-related potentials. Clin. Neurophysiol..

[42] Schoffelen J.M., Gross J. (2009). Source connectivity analysis with MEG and EEG. Hum. Brain Mapp..

[43] Tzourio-Mazoyer N., Landeau B., Papathanassiou D., Crivello F., Etard O., Delcroix N., Mazoyer B., Joliot M. (2002). Automated anatomical labeling of activations in SPM using a macroscopic anatomical parcellation of the MNI MRI single-subject brain. NeuroImage.

[44] Nunez P.L., Srinivasan R. (2006). Electric Fields of the Brain: The Neurophysics of EEG.

[45] van Diessen E., Numan T., van Dellen E., van der Kooi A.W., Boersma M., Hofman D., van Lutterveld R., van Dijk B.W., van Straaten E.C., Hillebrand A. (2015). Opportunities and methodological challenges in EEG and MEG resting state functional brain network research. Clin. Neurophysiol..

[46] Zalesky A., Fornito A., Bullmore E.T. (2010). Network-based statistic: Identifying differences in brain networks. NeuroImage.

[47] Xia M., Wang J., He Y. (2013). BrainNet Viewer: A network visualization tool for human brain connectomics. PLoS ONE.

[48] Krzywinski M., Schein J., Birol I., Connors J., Gascoyne R., Horsman D., Jones S.J., Marra M.A. (2009). Circos: An information aesthetic for comparative genomics. Genome Res..

[49] Acosta-Rodríguez V.A., Rijo-Ferreira F., Green C.B., Takahashi J.S. (2021). Importance of circadian timing for aging and longevity. Nat. Commun..

[50] Drummond S.P., Meloy M.J., Yanagi M.A., Orff H.J., Brown G.G. (2005). Compensatory recruitment after sleep deprivation and the relationship with performance. Psychiatry Res..

[51] Lowe C.J., Safati A., Hall P.A. (2017). The neurocognitive consequences of sleep restriction: A meta-analytic review. Neurosci. Biobehav. Rev..

[52] Praamstra P., Seiss E. (2005). The neurophysiology of response competition: Motor cortex activation and inhibition following subliminal response priming. J. Cogn. Neurosci..

[53] Fueggle S.N., Bucks R.S., Fox A.M. (2018). The relationship between naturalistic sleep variation and error monitoring in young adults: An event-related potential (ERP) study. Int. J. Psychophysiol..

[54] Lo J.C., Ong J.L., Leong R.L., Gooley J.J., Chee M.W. (2016). Cognitive Performance, Sleepiness, and Mood in Partially Sleep Deprived Adolescents: The Need for Sleep Study. Sleep.

[55] Goldman R.I., Stern J.M., Engel J., Cohen M.S. (2002). Simultaneous EEG and fMRI of the alpha rhythm. Neuroreport.

[56] Klimesch W., Sauseng P., Hanslmayr S. (2007). EEG alpha oscillations: The inhibition-timing hypothesis. Brain Res. Rev..

[57] Kaufmann T., Elvsåshagen T., Alnæs D., Zak N., Pedersen P.Ø., Norbom L.B., Quraishi S.H., Tagliazucchi E., Laufs H., Bjørnerud A. (2016). The brain functional connectome is robustly altered by lack of sleep. NeuroImage.

[58] Dai X.J., Liu C.L., Zhou R.L., Gong H.H., Wu B., Gao L., Wang Y.X. (2015). Long-term total sleep deprivation decreases the default spontaneous activity and connectivity pattern in healthy male subjects: A resting-state fMRI study. Neuropsychiatr. Dis. Treat..

[59] Honey C.J., Sporns O., Cammoun L., Gigandet X., Thiran J.P., Meuli R., Hagmann P. (2009). Predicting human resting-state functional connectivity from structural connectivity. Proc. Natl. Acad. Sci. USA.

[60] Murphy M., Riedner B.A., Huber R., Massimini M., Ferrarelli F., Tononi G. (2009). Source modeling sleep slow waves. Proc. Natl. Acad. Sci. USA.

[61] Kantarci K., Senjem M.L., Avula R., Zhang B., Samikoglu A.R., Weigand S.D., Przybelski S.A., Edmonson H.A., Vemuri P., Knopman D.S. (2011). Diffusion tensor imaging and cognitive function in older adults with no dementia. Neurology.

[62] Fransson P., Marrelec G. (2008). The precuneus/posterior cingulate cortex plays a pivotal role in the default mode network: Evidence from a partial correlation network analysis. NeuroImage.

[63] Coito A., Michel C.M., Vulliemoz S., Plomp G. (2019). Directed functional connections underlying spontaneous brain activity. Hum. Brain Mapp..

[64] Kreutzmann J.C., Havekes R., Abel T., Meerlo P. (2015). Sleep deprivation and hippocampal vulnerability: Changes in neuronal plasticity, neurogenesis and cognitive function. Neuroscience.

[65] Aron A.R., Fletcher P.C., Bullmore E.T., Sahakian B.J., Robbins T.W. (2003). Stop-signal inhibition disrupted by damage to right inferior frontal gyrus in humans. Nat. Neurosci..

[66] Chuah Y.M., Venkatraman V., Dinges D.F., Chee M.W. (2006). The neural basis of interindividual variability in inhibitory efficiency after sleep deprivation. J. Neurosci..

[67] Aron A.R., Robbins T.W., Poldrack R.A. (2014). Inhibition and the right inferior frontal cortex: One decade on. Trends Cogn. Sci..

[68] Bosch O.G., Rihm J.S., Scheidegger M., Landolt H.P., Stämpfli P., Brakowski J., Esposito F., Rasch B., Seifritz E. (2013). Sleep deprivation increases dorsal nexus connectivity to the dorsolateral prefrontal cortex in humans. Proc. Natl. Acad. Sci. USA.

[69] Vanni S., Tanskanen T., Seppä M., Uutela K., Hari R. (2001). Coinciding early activation of the human primary visual cortex and anteromedial cuneus. Proc. Natl. Acad. Sci. USA.

[70] Kong D., Soon C.S., Chee M.W. (2011). Reduced visual processing capacity in sleep deprived persons. NeuroImage.

[71] Zhao R., Zhang X., Fei N., Zhu Y., Sun J., Liu P., Yang X., Qin W. (2019). Decreased cortical and subcortical response to inhibition control after sleep deprivation. Brain Imaging Behav..

[72] Hodkinson D.J., O’Daly O., Zunszain P.A., Pariante C.M., Lazurenko V., Zelaya F.O., Howard M.A., Williams S.C. (2014). Circadian and homeostatic modulation of functional connectivity and regional cerebral blood flow in humans under normal entrained conditions. J. Cereb. Blood Flow Metab..

