# Supplementary material for: Decreased Functional Connectivity of Brain Networks in the Alpha Band after Sleep Deprivation Is Associated with Decreased Inhibitory Control in Young Male Adults
Source: Int J Environ Res Public Health. 2023 Mar 6;20(5):4663. doi: 10.3390/ijerph20054663 (PMC10002203; doi:10.3390/ijerph20054663)
Supplement: Supplementary file 1 [file ijerph-20-04663-s001.zip › ijerph-2225376-supplementary.pdf]

Table S1. 101 pairs of brain regions with significant functional connectivity.

Number	Brain region one	Brain region two
1	Frontal_Sup_Orb_L	Frontal_Mid_R
2	Frontal_Mid_R	Frontal_Mid_Orb_L
3	Frontal_Mid_Orb_L	Frontal_Mid_Orb_R
4	Frontal_Sup_Orb_L	Frontal_Inf_Tri_R
5	Frontal_Mid_Orb_L	Frontal_Inf_Tri_R
6	Frontal_Mid_Orb_R	Frontal_Inf_Tri_R
7	Frontal_Sup_Orb_L	Frontal_Mid_Orb_L
8	Frontal_Mid_R	Frontal_Mid_Orb_L
9	Frontal_Mid_Orb_L	Frontal_Mid_Orb_L
10	Frontal_Inf_Tri_R	Frontal_Mid_Orb_L
11	Frontal_Mid_R	Frontal_Mid_Orb_R
12	Frontal_Mid_Orb_L	Frontal_Mid_Orb_R
13	Frontal_Inf_Tri_R	Frontal_Mid_Orb_R
14	Frontal_Mid_R	Rectus_L
15	Frontal_Inf_Tri_R	Rectus_L
16	Cingulum_Post_L	ParaHippocampal_L
17	Olfactory_L	Calcarine_L
18	Rectus_L	Calcarine_L
19	Cingulum_Post_L	Calcarine_L
20	Cingulum_Post_R	Calcarine_L
21	Cingulum_Post_L	Calcarine_R
22	Cingulum_Post_R	Calcarine_R
23	Hippocampus_R	Calcarine_R
24	Calcarine_L	Calcarine_R
25	ParaHippocampal_L	Cuneus_L
26	Calcarine_L	Cuneus_L
27	Calcarine_R	Cuneus_L
28	Calcarine_R	Cuneus_R
29	Cingulum_Post_L	Lingual_L
30	Cingulum_Post_R	Lingual_L
31	Calcarine_L	Lingual_L
32	Calcarine_R	Lingual_L
33	Cuneus_L	Lingual_L
34	Cuneus_R	Lingual_L
35	Cingulum_Mid_L	Lingual_R
36	Cingulum_Post_L	Lingual_R
37	Cingulum_Post_R	Lingual_R
38	Cuneus_L	Lingual_R
39	Calcarine_L	Occipital_Sup_L
40	Precentral_L	Occipital_Mid_L
41	Cingulum_Post_L	Occipital_Mid_L

42	Calcarine_L	Occipital_Mid_L
43	Cuneus_L	Occipital_Mid_L
44	Lingual_L	Occipital_Mid_L
45	Lingual_L	Occipital_Mid_R
46	Cingulum_Mid_L	Occipital_Inf_L
47	Cingulum_Post_L	Occipital_Inf_L
48	Cingulum_Post_R	Occipital_Inf_L
49	Cuneus_L	Occipital_Inf_L
50	Occipital_Sup_L	Occipital_Inf_L
51	Occipital_Mid_L	Occipital_Inf_L
52	Olfactory_L	Occipital_Inf_R
53	Cingulum_Mid_L	Occipital_Inf_R
54	Cingulum_Post_L	Occipital_Inf_R
55	Cingulum_Post_R	Occipital_Inf_R
56	Cuneus_L	Occipital_Inf_R
57	Occipital_Sup_L	Occipital_Inf_R
58	Occipital_Mid_L	Occipital_Inf_R
59	Occipital_Inf_L	Occipital_Inf_R
60	Cingulum_Post_L	Fusiform_L
61	Cuneus_L	Fusiform_L
62	Cingulum_Post_L	Fusiform_R
63	Cingulum_Post_R	Fusiform_R
64	Cuneus_L	Fusiform_R
65	Calcarine_L	Postcentral_L
66	Lingual_L	Postcentral_L
67	Occipital_Mid_L	Postcentral_L
68	Occipital_Inf_L	Postcentral_L
69	Occipital_Inf_R	Postcentral_L
70	Cingulum_Post_L	Postcentral_R
71	Occipital_Inf_R	Parietal_Inf_L
72	Frontal_Mid_Orb_R	Parietal_Inf_R
73	Frontal_Mid_Orb_R	Parietal_Inf_R
74	Calcarine_L	Precuneus_L
75	Lingual_L	Precuneus_L
76	Occipital_Inf_L	Precuneus_L
77	Occipital_Inf_R	Precuneus_L
78	Postcentral_L	Paracentral_Lobule_L
79	Precentral_L	Paracentral_Lobule_R
80	Postcentral_L	Paracentral_Lobule_R
81	Lingual_R	Temporal_Sup_R
82	Occipital_Inf_R	Temporal_Sup_R
83	Fusiform_R	Temporal_Sup_R
84	Olfactory_L	Temporal_Mid_L
85	Cingulum_Post_L	Temporal_Mid_L

86	Calcarine_L	Temporal_Mid_L
87	Cuneus_L	Temporal_Mid_L
88	Calcarine_L	Temporal_Mid_R
89	Lingual_L	Temporal_Mid_R
90	Lingual_R	Temporal_Mid_R
91	Occipital_Inf_R	Temporal_Mid_R
92	Fusiform_R	Temporal_Mid_R
93	Parietal_Inf_R	Temporal_Mid_R
94	Temporal_Mid_R	Temporal_Inf_L
95	Rectus_L	Temporal_Inf_R
96	Rectus_R	Temporal_Inf_R
97	Cingulum_Post_L	Temporal_Inf_R
98	Calcarine_L	Temporal_Inf_R
99	Occipital_Inf_R	Temporal_Inf_R
100	Temporal_Sup_R	Temporal_Inf_R
101	Temporal_Mid_R	Temporal_Inf_R
